# Cryptic Disc Structures Resembling Ediacaran Discoidal Fossils from the Lower Silurian Hellefjord Schist, Arctic Norway

**DOI:** 10.1371/journal.pone.0164071

**Published:** 2016-10-26

**Authors:** Christopher L. Kirkland, Breandán A. MacGabhann, Brian L. Kirkland, J. Stephen Daly

**Affiliations:** 1 Department of Applied Geology, (Centre for Exploration Targeting – Curtin Node and Core to Crust Fluid Centre), Curtin University, Perth, Australia; 2 Department of Geography, Edge Hill University, Ormskirk, Lancashire, England; 3 Independent Researcher, Coleraine, N. Ireland; 4 UCD School of Earth Sciences and UCD Earth Institute, University College Dublin, Dublin, Ireland; Université de Poitiers, FRANCE

## Abstract

The Hellefjord Schist, a volcaniclastic psammite-pelite formation in the Caledonides of Arctic Norway contains discoidal impressions and apparent tube casts that share morphological and taphonomic similarities to Neoproterozoic stem-holdfast forms. U-Pb zircon geochronology on the host metasediment indicates it was deposited between 437 ± 2 and 439 ± 3 Ma, but also indicates that an inferred basal conglomerate to this formation must be part of an older stratigraphic element, as it is cross-cut by a 546 ± 4 Ma pegmatite. These results confirm that the Hellefjord Schist is separated from underlying older Proterozoic rocks by a thrust. It has previously been argued that the Cambrian Substrate Revolution destroyed the ecological niches that the Neoproterozoic frond-holdfasts organisms occupied. However, the discovery of these fossils in Silurian rocks demonstrates that the environment and substrate must have been similar enough to Neoproterozoic settings that frond-holdfast bodyplans were still ecologically viable some hundred million years later.

## Introduction

The Neoproterozoic Era includes the oldest known macroscopic fossil communities [1–5], including some suggested to have been early animals [6–13] and extinct lineages [14–17]. Discoidal impressions account for much of the preserved record of this life [18], but similar fossils are comparatively rare in the Phanerozoic, in which the only reported examples are Cambrian in age [19,20].

**Fig 1:**
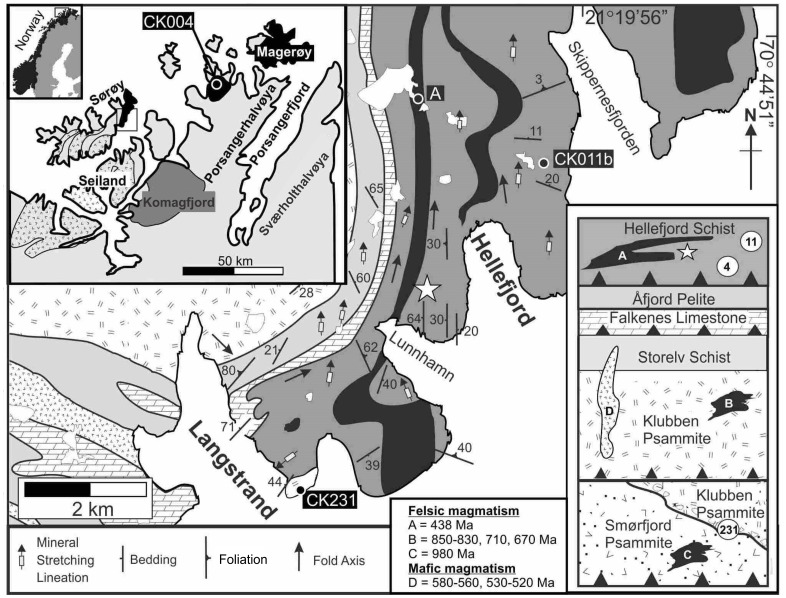
Locality and stratigraphy of the fossils. Geological map of the Langstrand—Hellefjord area, Sørøy (modified after [21]). Fossil locality indicated by star. Inset left: main tectonic units of Finnmark with overview map of Norway. Inset right: simplified tectonostratigraphy of KNC and overlying Hellefjord Schist.

Here, we report the occurrence of discoidal fossils of Silurian age, which although simple, appear apparently indistinguishable in morphology from examples of Ediacaran age. Like all discoidal impressions, these markings require caution in interpretation, since they are simple in form. The fossils described in this article occur in the Hellefjord Schist Formation, within the Norwegian Caledonides in Finnmark, Arctic Norway (Fig 1). This unit contains volcaniclastic horizons and is cut by several granitoid sheets that precisely constrain its depositional age to the mid-Llandovery (early Silurian). Exposures of interbedded psammite-pelite reveal discoidal impressions that are morphologically indistinguishable from Ediacaran examples *Nimbia* and *Tirasiana*. The depositional environmental for this unit is interpreted as moderate to deep-water pelagic sediments intercalated with distal turbidites, and includes volcanic outfall [21,22]. Here we use new field observations in conjunction with a SIMS U-Pb geochronology dataset in an effort to better constrain the temporal range of discoidal fossils and to refine the stratigraphy of the Arctic Caledonides.

## Geological Setting

The Hellefjord Schist was originally considered a component of the Kalak Nappe Complex (KNC), a tectonically assembled sequence of overthrust units in the Norwegian Caledonides [23]. However, more recent work demonstrates the Hellefjord Schist is a component of the overlying Magerøy Nappe, a package of metasedimentary rocks intruded by gabbroic and granitic bodies of Silurian age [21,24]. The underlying basement to the Hellefjord Schist, the KNC, was initially regarded as constructed from a single, conformable package, the Sørøy Succession, which comprises (metamorphosed) shallow marine siliciclastic rocks, limestone, deeper marine pelagic rocks and turbidites [22,25,26]. However, U—Pb dating of granitic intrusions and detrital zircons indicates that the KNC metasediments form several age-distinct lithotectonic packages [27,28].

The rocks in the lower nappes of the KNC (Svaerholt Succession) have a depositional age of c. 980–1030 Ma [27] and were affected by a c. 980 Ma tectonomagmatic event [29]. Unconformably overlying the Svaerholt Succession is the Sørøy Succession, which consists of the Klubben Psammite and Storelv Schist that were deposited between c. 840 and 910 Ma [27]. On the island of Sørøy, a sequence of metamorphosed limestone and pelite (the Falkenes Limestone and Åfjord Pelite) overlies the Sørøy Succession (Fig 1). Chemostratigraphy on the carbonate rocks suggests a depositional age of 760–710 Ma, with the contact to underlying units interpreted to be tectonic [30].

The Hellefjord Schist was originally regarded as the youngest component of the KNC [22,31]. It is intruded by 438 ± 2 Ma granitoid sheets and contains detrital zircons as young as 438 ± 4 Ma [21]. These temporal constraints are consistent with correlation of the Hellefjord Schist with the fossiliferous Middle Llandovery Juldagnes Formation within the Magerøy Nappe [32–35]. The Magerøy Nappe is recognized as a structure that developed during the c. 420 Ma Scandian Orogeny [36]. Krill and Zwaan [37] linked the Magerøy Nappe to the KNC suggesting deformation occurred during the Scandian throughout these units a finding consistent with Ar-Ar geochronology [38].

### Lithological characteristics of the Hellefjord Schist and its regional correlation

The Hellefjord Schist crops out extensively over NE Sørøy, but is also found in limited exposures on Porsangerhalvøya [36] (Fig 1). It is a monotonous sequence of medium- to fine-grained quartz-, plagioclase-, amphibole- and biotite-bearing schist (psammite) with finer-grained biotite-, quartz-, and plagioclase-bearing phyllite (pelite) [22]. Pelitic beds contain garnet, and the unit has been metamorphosed to amphibolite facies. Sedimentary structures include load casts and flame structures, with rare current ripples indicating flow from the NNW [22]. The Hellefjord Schist reflects a flysch deposit in that it is a sequence of deep marine sedimentary rocks deposited in a back-arc during an early stage of orogenesis [21]. At the contact with the underlying Falkenes Limestone, the Gamnes Conglomerate is traditionally considered as a primary basal feature of the Hellefjord Schist [22,39].

The Hellefjord Schist is intruded by c. 438 Ma granites that are compositionally similar to coeval granites within Laurentian-derived allochthonous units elsewhere in the Norwegian Caledonides [21,24]. Similar metasedimentary rocks on Magerøy are intruded by c. 438 Ma gabbros [40], from which paleomagnetic constraints imply an equatoral position on the Laurentian margin during the Silurian [24]. These mafic intrusions reflect development of volcanic arcs and back-arcs in the northern Appalachian segment of the margin of Laurentia [41].

## Fossils in the Hellefjord Schist

**Fig 2:**
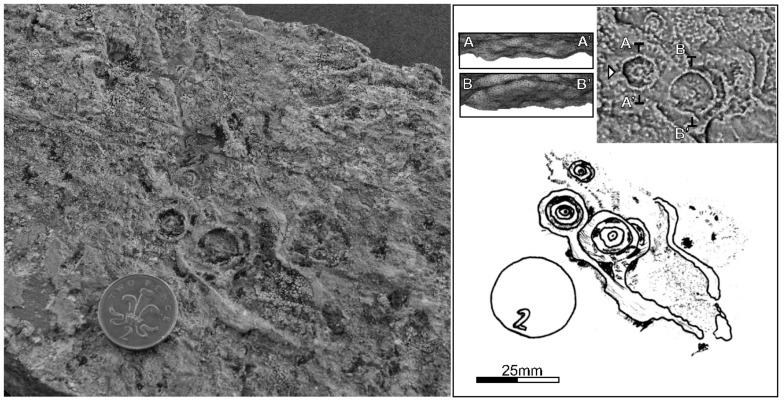
Images of the Hellefjord fossils. Left: photograph of discoidal fossils and sinusoidal tube casts, Hellefjord Schist. Right: sketch of salient features of fossils. Inset: photogrammetric surface reconstruction with radiance scaling shader. Planes A-A’ and B-B’ reflect eastward looking tilted section through discoids.

Several discoidal positive hyporelief casts have been found on the lower bedding surface of steeply dipping pelite—psammite interbeds of the Hellefjord Schist at Pikfjellet, on northern Sørøy (gravity cast-style preservation; Fig 2). The rock in which the fossils are found is fine-grained and metamorphic grain size coarsening has not significantly affected this specific unit. In addition it lacks a pervasive metamorphic foliation. A 3D photogrammetric reconstruction of the material is provided in S1 Video. These specimens are characterized by a depressed, low-relief disc, 2–20 mm in diameter, with a central boss that is less than one tenth of the diameter of the whole disc. The periphery of the disc is a raised rim with a sharp outer and inner edge up to 5 mm apart. Between the rim and the boss, the disc is either smooth or ornamented by faint concentric rings. On some discs, faint radial grooves occur between the ring edges (Fig 2). The relief profile of the larger discs concurs with the general description of *Tirasiana*; a concentric annulus surrounding a prominent tubercle [42–45]. The larger discs preserve two concentric rings. The smaller discs also have a central tubercle but are enclosed by a single circular ridge and match descriptions of *Nimbia* [42,43,46]. The density of discs on any surface is patchy, with local crowding in the form of chains, where several discs cluster along a direction parallel to the dominant orientation of tube-like features. Overlapping borders are not observed. No obvious organic material is associated with the structures.

The surface containing the discodial impressions also preserves several sinusoidal tube like casts (Fig 2). These tube like structures are up to 5.7 mm wide and can extend for up to 90 mm, describing distinct meanders. The sinusoidal meanders are suggestive of a path avoiding the periphery of other now poorly preserved discoidal fossils. Segments of the tube casts closest to the discoidal fossils appear to lie in the same general southerly direction. These apparent tubes are challenging to interpret but trend towards the discoidal fossils, though do not overprint them. This may imply an association of the discs and tube structures with attachment between the two elements; that is, the discs may represent holdfasts.

The preservation of these discoidal fossils is similar to Fermeuse-style preservation of fossils of Ediacaran age in, for example, the Fermeuse Formation of Newfoundland [47], the Innerelv Member of the Stappogiedde Formation of Norway [48], and the Coomb Formation of Wales [49,50]. Narbonne [51] defined this style of fossil assemblage preservation as typically preserving only trace fossils and the bases of holdfasts, later restated less restrictively by MacGabhann [43] as assemblages in which all the fossils are ‘gravity casts’ (positive hyporelief casts and/or negative epirelief molds). In this style of preservation, the attachment between stems and holdfasts could not be preserved, as the attachment point lies directly above the basal surface of the holdfast. Nonetheless, it is feasible that these tube-like structures represent poorly preserved body fossils of collapsed stalks influenced by current motion [52]. Due to the lack of preservation of more complex frondose elements, if they existed, any interpretation must remain tentative for these tubes.

## Temporal Constraint on the Hellefjord Schist

In order to verify the age of the Hellefjord Schist, U-Th-Pb SIMS geochronology was performed on a sample from the same psammite horizon that yielded a limited 438 ± 4 Ma volcaniclastic zircon population [21]. In addition we report the age of detritus within a pelitic sample from the Hellefjord Schist at Bakfjorden, on Porsangerhalvøya (Fig 1), to assess the lateral continuity of this purported volcaniclastic component. To provide additional constraints on the stratigraphy, we also determined the age of a pegmatite intruding the Gamnes conglomerate. The analytical method (S1 Text) and data table (S1 Table) are provided as Supporting Information.

### Volcaniclastic psammite, Hellefjord Schist, Sørøy

CK011b was collected 1.1 km NE of Hellefjord on Sørøy (Fig 1). The sample was recovered from a laterally continuous psammite within a dominantly pelitic succession. This sample yielded only a few zircons that range from colourless to pale yellow. They are up to 100 μm long with aspect ratios of 5:1 or less. In cathodoluminescence (CL) images, most grains display concentric growth zoning or sector zoning. In some cases the zonation is truncated at grain edges. Many grains have rounding of terminations consistent with mechanical abrasion during transport.

**Fig 3:**
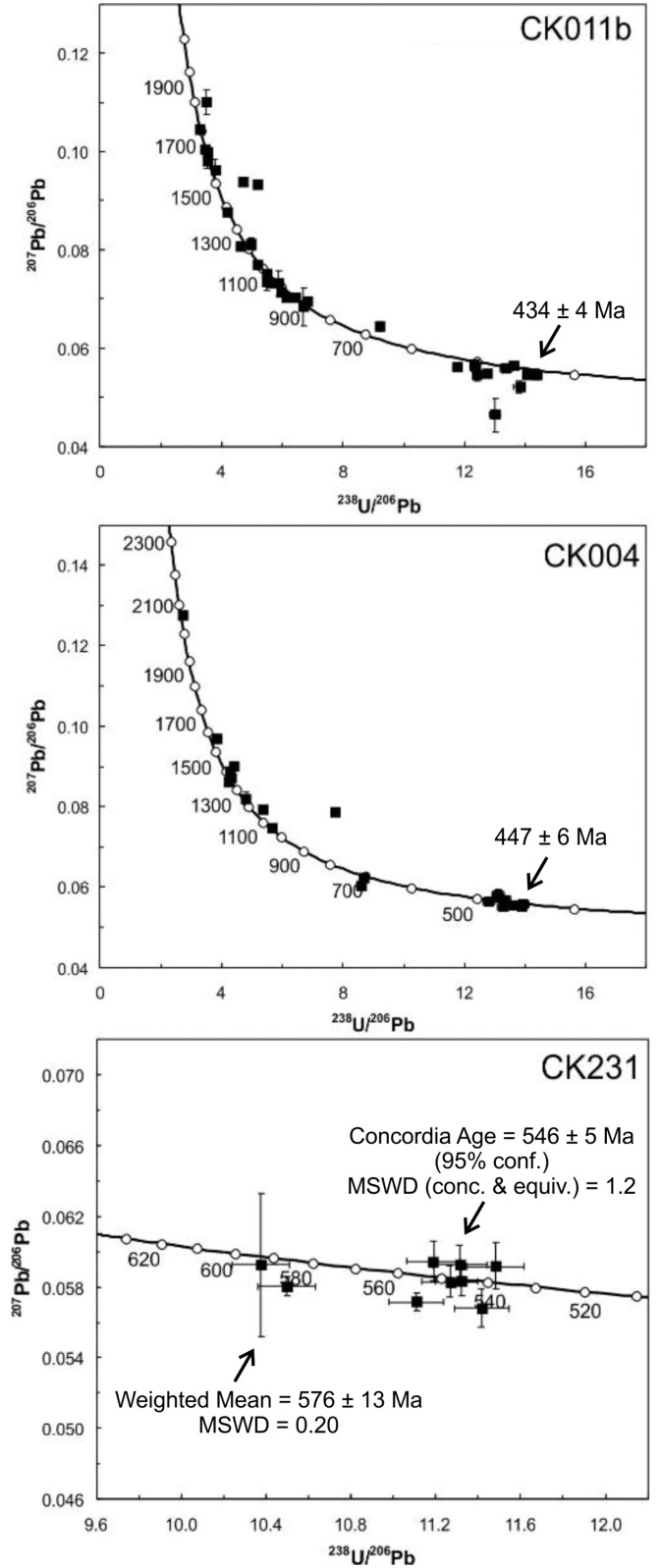
Inverse concordia diagrams for zircon grains analysed by SIMS. CK011 and CK004 are detrital material. The age of the youngest analysis is shown. CK231 is interpreted to reflect both inheritance and a magmatic population. U—Pb data are plotted as 2 σ error crosses.

Thirty-three analyses were obtained from 23 zircon grains, with all but five analyses within 2σ uncertainty of concordia (Fig 3). Five discordant analyses are not considered further. The youngest analysis from the centre of a euhedral idiomorphically-zoned crystal yields a 207-corrected ^238^U/^206^Pb age of 434 ± 4 Ma (1σ). The youngest age probability peak that includes contributions from three analyses is 502 Ma. Other detrital age peaks are defined at 886 Ma (3 analyses), 934 Ma (3 analyses), 971 Ma (4 analyses), 1030 Ma (4 analyses), and 1626 Ma (4 analyses).

### Pelite, Hellefjord Schist, Porsangerhalvøya

CK004 is a semipelite collected on a roadside outcrop at Skihsbukta, at the head of Bakfjorden (Fig 1). This sample yielded a very small population of predominantly small colourless zircon grains, most of which are < 20 μm long with aspect ratios up to 5:1. In CL images, most grains display well developed oscillatory zonation. Several grains are mantled with a thin sub 2 μm high CL-response zircon overgrowth.

Nineteen analyses were obtained on nineteen zircon crystals. Five analyses are outside 2σ uncertainty of concordia and are not considered further. The youngest analysis from the centre of a euhedral idiomorphically zoned crystal yields a 207-corrected ^238^U/^206^Pb age of 447 ± 6 Ma (1σ; Fig 3). The youngest age probability peak is at 451 Ma defined by contributions from three analyses. A secondary age probability peak is defined at 1357 Ma, also by three analyses.

### Pegmatite, Gamnes Conglomerate, Sørøy

CK231 was sampled from a pegmatite that cross-cuts both bedding and a metamorphic lineation within the Gamnes Conglomerate on Sørøy (Fig 1). The conglomerate has a matrix dominated by quartz, feldspar and biotite, in which clasts are dominated by psammite [53]. Zircons from this sample fall into two morphological categories. The dominant population consists of euhedral stubby prisms with aspect ratios up to 4:1. The crystals are up to 300 μm long and under CL most have a homogeneous low response, with some grain cores showing faint oscillatory or convoluted zonation. A minor population consists of small rounded grains less than 20 μm in length that have a homogeneous dark CL response.

Nine analyses were obtained from eight grains. All analyses are within uncertainty of Concordia (Fig 3). Seven analyses from six grains yield a Concordia age of 546 ± 4 Ma (MSWD = 1.2), interpreted as the age of magmatic crystallization based on the zircon CL texture and grain morphology. Two analyses of rounded zircons yield a weighted mean ^238^U/^206^Pb age of 576 ± 13 Ma (MSWD = 0.2), interpreted as the age of inherited components.

## Discussion

### Stratigraphic implications

Two new samples of the Hellefjord Schist confirm its young volcaniclastic cargo. Considering all U-Pb data on the volcaniclastic psammite from Sørøy [21] the five youngest zircon grains yield a Concordia age of 436 ± 5 Ma (MSWD = 1.2), which serves as a maximum age of deposition. The youngest coherent zircon age component from the pelite on Porsangerhalvøya yields a concordia age of 449 ± 7 Ma (MSWD = 1.1). Given the well-constrained age of granites intruding the Hellefjord Schist of 438 ± 2 Ma [21] and the analytical overlap of this date with the constraint on sedimentation (which must be older), a Monte Carlo approach can be used along with the analytical uncertainty to increase the precision of the dates [54]. This approach suggests deposition of the Hellefjord Schist occurred between 437 ± 2 and 439 ± 3 Ma, also constraining the age of the discoidal features found within it.

Discoidal impressions of a similar morphology and size distribution to the specimens from the Hellefjord Schist on northern Sørøy have also been reported from farther south on Sørøy [55]. These have been subject to controversy, both in terms of their stratigraphic assignment and their phylogenetic placement. Although originally attributed to archaeocyathids [55], this interpretation was subsequently discredited [56]. Nevertheless, a biological origin for the discoidal structures was not rejected. The stratigraphic assignment of the host metasedimentary unit for these fossils on southern Sørøy is uncertain, with it being variably assigned to the Klubben Psammite or Falkenes Limestone. Additionally, the southern Sørøy unit has been described as a metasedimentary raft within the c. 570 Ma Storelv Gabbro, implying that the discoidal impressions are older than the Cambrian [57]. However, Krill and Zwaan [37] questioned the nature of the contact between the fossiliferous unit and basement, highlighting that it may be tectonic.

The Gamnes Conglomerate [22] has been regarded as a basal component of the Hellefjord Schist Formation, prompting notions of an original basement-cover relationship with the subjacent Falkenes Limestone and Åfjord Pelite. Such a relationship would support the idea that the Magerøy Nappe was a younger but nonetheless integral part of the KNC, as opposed to a unit juxtaposed with the KNC during c. 420 Ma Scandian thrusting. The pegmatite intruding the Gamnes Conglomerate (CK231) yields a crystallization age of 546 ± 4 Ma, which indicates that the host conglomerate must be older and cannot be a basal component of the Hellefjord Schist. Furthermore, we note a lithological and textural similarity of the Gamnes Conglomerate to the basal conglomeratic unit of the Klubben Psammite as preserved on Hjelmsøy [28].

Ediacaran magmatic activity in the KNC is widespread, with the emplacement of gabbro, pyroxenite, diorite, granite, and syenite in the Seiland Igneous Province between about 580 and 560 Ma [57]. A later phase of alkaline magmatic activity is represented by nepheline syenite pegmatites with U-Pb dates of 530–520 Ma [58]. It would seem logical that inheritance of Seiland Igneous Province zircons into the pegmatite accounts for the c. 580 Ma xenocrystic zircon crystals. These ages are also similar to Grenvillian basement in the Central Appalachians, which was affected by episodes of granitic magmatism (A-type) at 765 to 680 Ma and 620 to 550 Ma, with extensive mafic volcanism at 570 to 560 Ma attributed to rifting of Laurentia [59].

The Gamnes pegmatite dated at 546 Ma cuts a N—S lineation in the surrounding country rock, indicating a deformation phase prior to this time affecting the conglomerate. In the overlying Hellefjord Schist, a similar N—S lineation has been constrained to a period of Scandian lateral escape between 431–428 Ma [53]. However, the new U-Pb age for the Gamnes pegmatite requires pre-Scandian high strain deformation whose lineation is now subparallel to the Scandian fabric that is at least 115 Ma younger. Biotite clots from the Gamnes Conglomerate yield an Ar—Ar cooling age of 401 ± 7 Ma [27], and undeformed muscovite vugs within the Gamnes pegmatite yield an Ar-Ar cooling age of 418 ± 6 Ma [53]. The published Ar-Ar results constrain deformation to before 418 Ma, consistent with the zircon U-Pb age for the pegmatite, which indicates deformation prior to 546 ± 4 Ma. Additionally, the Ar-Ar results suggest uplift and cooling through muscovite and biotite closure temperatures after Scandian thrusting. The age and deformation history indicated for the Gamnes Conglomerate demonstrates it cannot be a component of the Hellefjord Schist, removing a significant argument for a primary basement-cover relationship between the Hellefjord Schist and the KNC.

The Hellefjord Schist has been conclusively correlated to the fossiliferous Juldagnes Formation on the nearby island of Magerøy based on both age [21,24] and petrographic similarity [35]. The Juldagnes Formation represents a flysch sequence of turbidites [60,61] and is underlain by the Nordvågen Group of pelites with local occurrences of conglomerate, limestone, quartzite and greywacke. The Nordvågen Group has been interpreted to contain a gradually shallowing sequence, whereas the overlying Juldagnes Formation represents a period of basin deepening [60]. Fossils within the Nordvågen Group include Early Silurian crinoids, pentameride brachiopods, favositids, halysitids, heliolitids, and rugose corals [33,60]. In contrast the Juldagnes Formation contains a deeper water Early Silurian assemblage of ichnofauna (*Protopalaeodictyon* and *Scolicia plana*) and monograptides (*monograptus sandersoni*) [60,62].

### Discoidal impressions within the Hellefjord Schist

#### Biogenic origin for the fossils

The first priority must be to establish whether the Silurian discs are indeed biological in origin, as discoidal structures may be formed by inorganic processes, including raindrop imprints [63], fluid escape structures (sand volcanoes), gas escape structures [64], load casts [65], salt pseudomorphs [66], and pyrite rosettes [67].

Salt pseudomorphs and pyrite rosettes may be immediately ruled out on morphological grounds, as they show neither the radial structures characteristic of pyrite rosettes [67], nor the collapse structures typical of salt pseudomorph pseudofossils [66].

Raindrop impressions [63] may similarly be ruled out, as the discs characteristically do not include annuli within the pit formed by raindrop impact. The Sørøy discs, at up to 20mm, are also considerably larger than the maximum size of raindrop impressions. Likewise, gas escape structures, which are often commonly mistaken for raindrop impressions [64], are generally considerably smaller than the maximum size of the Sørøy specimens.

Liquefaction or fluid escape structures such as load casts, or so-called sand volcanoes, are formed due to liquefaction [65] following rapid deposition of water-rich sediment [68] or during/after earthquakes [69]. However, the cross sectional morphology of the discoidal fossils in the Hellefjord Schist is inconsistent with a load cast or sand volcano, given that multiple concentric annuli are preserved that undulate to an extent greater than the central region. The narrow stem-like feature running towards the central bosses are dissimilar to sheet flow on the edge of a sand volcano, which would be expected to diverge away from its vent. Additionally, there is no interaction between discoidal fossils where one might expect irregularity to be developed when multiple sand volcanoes occur within a confined area, and there is no indication of a vertical fluid escape structure in the centre of the discs in cross section. In summary, an abiotic origin seems unlikely.

Partly or wholly biological processes may produce discoidal structures, including scratch circles [70], bacterial colonies [71] and water or gas escape from microbial mats [72,73]. The surface expression of vertical burrows may also produce discoidal structures.

Vertical burrow trace fossils may be ruled out due to the lack of vertical pipes in cross-section (Fig 2), as may fluid escape in concert with a microbial mat, which the lack of wrinkling and crack-fill also argues against. Gas escape through a microbial mat also appears unlikely due to the lack of such wrinkling and crack-fill [72], in addition to the presence of annuli and apparent stems.

Microbial colonies can produce discoidal structures of similar size and shape to the Hellefjord discoidal fossils [71,74]. However, the widely-spaced and sharp nature of the annuli of the Sørøy specimens would be very unusual for a microbial colony, which tend to have multiple closely-spaced concentric annuli.

Scratch circles form when a tethered organism is rotated by currents, with the upper parts of the organisms dragged on the substrate surface around the attachment point, leaving arcuate to circular marks on the sediment—water interface [70,75]. Radial impressions can also be left by the stalk. However, the Hellefjord discs are unlikely to be scratch circles, as the sharp nature of the annuli would again be unusual in such an interpretation—scratch circles tend to have rings with smoother edges due to the erosional mode of formation. The apparent stalk of the Sørøy specimens is also far larger than the disc radius, with a scratch circle interpretation therefore requiring the stalk only to have been in contact with the substrate in the immediate vicinity of the attachment point, which is biomechanically unlikely. It is also worth noting that a scratch circle interpretation for the discs would indicate the presence of organisms with near-identical morphology and ecology to those envisaged by a biogenic interpretation. In our view a fully biogenic interpretation of the discs is the most parsimonious interpretation.

#### Phylogeny and relationships

Discoidal fossils are most commonly associated with fossil localities of Neoproterozoic age [43]. Initially regarded as jellyfish impressions [76–81], it is now understood that Neoproterozoic discoidal impressions can be formed by a wide range of benthic discoidal organisms [43], including—but not limited to—microbial colonies [71], fungi [82], and cnidarians [43,83]. Multiple lineages of epibenthic frondose Neoproterozoic organisms, such as rangeomorphs and arboreomorphs, also produced discoidal impressions through a basal flattened or bulbous disc which acted as a holdfast for an upper stem and petalodium [18,84–91].

Discoidal structures are also known from post-Ediacaran sediments. Concentrically structured discoidal fossils comparable to *Nimbia* and *Tirasiana* have been reported from the lower Cambrian of California [20] and from the Digermul Peninsula, northern Norway [19]. Younger discoidal fossils were produced by a wide range of organisms, arguably even wider than those of Neoproterozoic specimens, including the extinct fossil eldonids [92,93] and a number of *incertae sedis* organisms such as *Patanacta pedina*, *Parasolia actiniformis*, or *Velumbrella bayeri* [94–96] in addition to extant clades like cnidarians [97,98]. However, these are generally different in aspect to Neoproterozoic discoidal remains.

Other Phanerozoic discoidal structures are known to have been produced abiogenically [99,100]. Scratch circles in particular are known throughout the Phanerozoic, including specimens from the Cambrian of Ireland [18,70] originally assigned to *Nimbia* by Crimes et al. [101], and examples from the Paleocene of Italy produced by foraminifera [102].

The discoidal forms in the Hellefjord Schist do not have the complexity of phylogenetically determinate Phanerozoic discoidal organisms such as eldonids or cnidarians, nor do they resemble any of the *incertae sedis* material. Rather, they cannot be distinguished from Neoproterozoic discoidal taxa, and would be identified variously as *Nimbia* or *Tirasiana* if found in sediments of Ediacaran age.

Due to the morphological simplicity of discoidal structures, the range of discoidal organisms, and the potential for taphonomic processes to cause variation between the preserved forms of similar organisms, identifying the phylogenetic origin of discoidal fossils is commonly difficult. This is especially true in the Neoproterozoic, with numerous extinct lineages existing alongside the ancestors of extant discoidal organisms. In addition, structural elements within the water column, including more delicate frondose structures, are far more difficult to preserve than body parts within or on the substrate [51,103,104]. As a result, it may be impossible to identify whether or not discoidal structures are holdfasts of epibenthic frondose organisms.

The available evidence from the Hellefjord fossils is consistent with their genesis under taphonomic processes similar to those responsible for the preservation of discoidal gravity casts in Ediacaran sediments. Such Fermeuse-style assemblages preserve only gravity-cast fossils (in positive hyporelief on bed soles), such that only features that were on the base of the organisms, in contact with the substrate, can be preserved. Hence, attachments between a disc and its stem can never be recorded by this style of preservation. However, in some occurrences a stem impression may emanate from the margin of the disc, as potentially hinted at by the Hellefjord specimens. The Hellefjord organisms were therefore apparently at least similar in general morphology to Ediacaran-aged stem-holdfast organisms, with a stalk extending from a basal discoidal attachment to the substrate.

The absence of any biomineralisation in the Hellefjord discs suggests a soft-bodied nature for the producing organisms. The preservation of imprints or traces from soft-bodies necessitates a general lack of heavy bioturbation [92]. As in the case of many Ediacaran sites, certain body elements may not have been preserved due to removal in the water column or labile tissue destruction prior to complete lithification [104].

The observations from the Hellefjord Schist extends the stratigraphic range of similar fossils to the Silurian and cautions about the simple nature of certain discoidal forms. The Hellefjord Schist and correlative Juldagnes Formation were deposited in a deep marine slope setting as evidenced by the lithofacies, ichnofacies and fossil assemblage implying a relatively shore-distal environment, mainly receiving low velocity turbidity currents. Such a deep water setting is consistent with the habitat of frond-holdfast organisms of Ediacaran age, which are known ranging from deep-marine basinal contour-current and turbidite settings to shoreface environments above fair-weather wave base [47,105–107].

**Fig 4:**
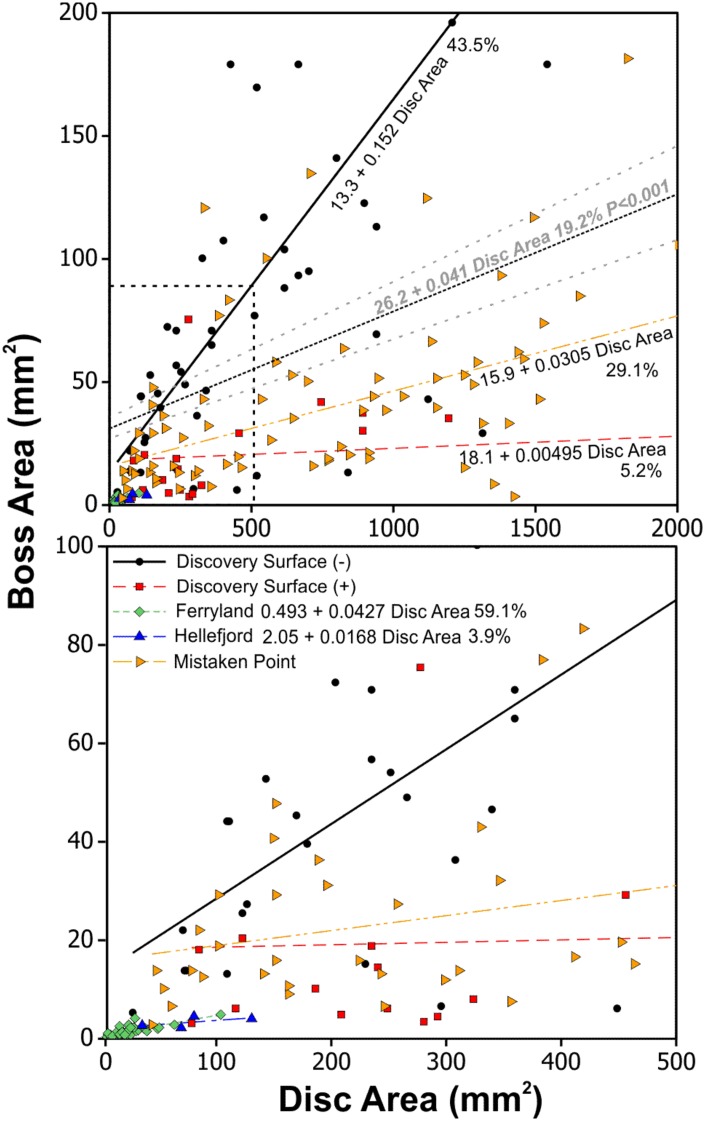
Boss area versus disc area with linear regression fits to all data and by geographic location. The adjusted R^2^ value is shown as a percentage for each fit and indicates the degree of scatter accounted for by the regression. Upper plot shows 0–2000 and 0–200 mm^2^ region only. Lower plot is enlargement of dashed region (Newfoundland data from [108]). − = negative epirelief; + = positive epirelief.

**Fig 5:**
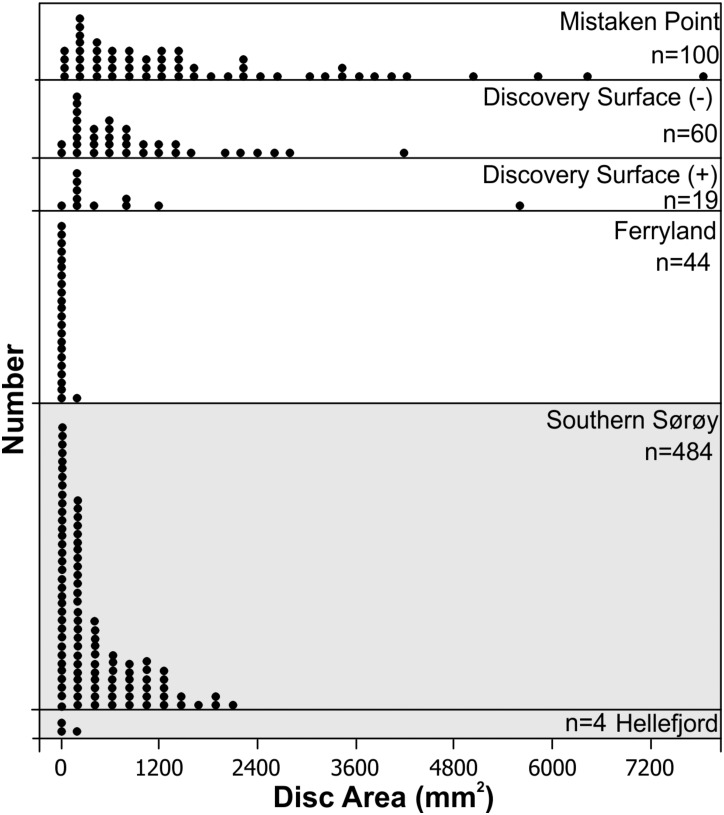
Plot of disc area versus number of measurements from Ediacaran sites in Newfoundland compared to those from Sørøy, northern Norway. Each symbol represents up to two observations. Measurements include results from Burzynski and Narbonne [108] and Holland and Sturt [55]. The disc area for the Sørøy sites is most similar to Fermeuse-style positive hyporelief fossils at Ferryland.

**Fig 6:**
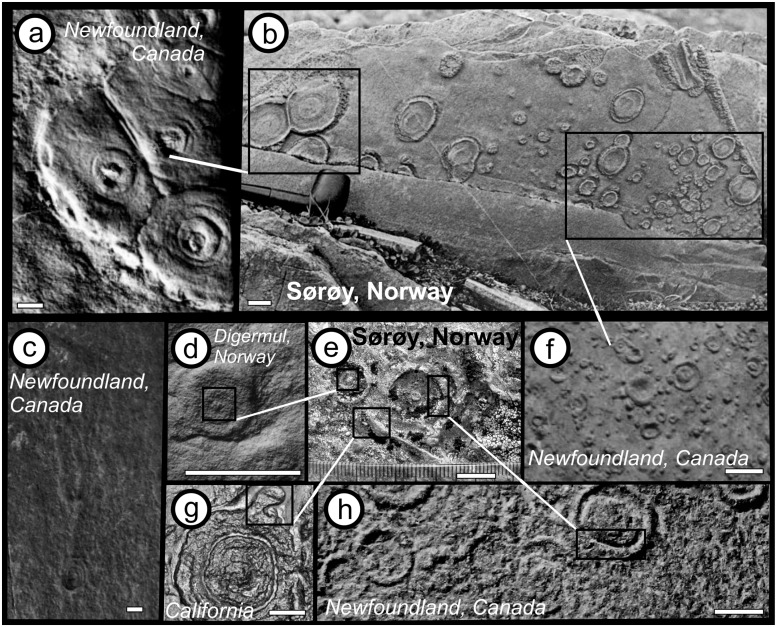
Examples of preservation styles found in Ediacaran (and one early Cambrian) sites compared to fossils from Sørøy, northern Norway. A: Cluster of flat-convex discs from Newfoundland [84]; note similarity in contact between discs and those in B. B: Southern Sørøy discs of varying size [55]. C: *Primocandelabrum* from Newfoundland showing holdfast and branching stem which may have shared some similar morphological elements to the Hellefjord Schist forms. D: Disc feature from Digermul Peninsula, Norway [19]. Note similarity of central boss to E. E. Discs and tube cast (stem) from Hellefjord Schist Sørøy—see Fig 2. F: Small discs (Type morph of *Aspidella*) showing central invagination with recessed bosses [84] note similarity to areas on B. G: Early Cambrian fossil from California, previously compared to discs of Ediacaran age, highlighted region with “burrow” abutting disc [20] note similarity to E. H: Positive rimmed disc impressions associated with *Aspidella*, Newfoundland [83], note similar edge morphology to E. Scale bars are 1 cm.

The relationship between disc size and central boss size has been investigated for discoidal fossils of Ediacaran age in Newfoundland by Burzynski and Narbonne [108]. They observed a positive relationship between disc diameter and boss diameter, consistent with biological dimensions where a larger holdfast would be required to support a larger stem and other appendages (Fig 4). Using the Burzynski and Narbonne [108] dataset along with that from the Hellefjord Schist indicates a statistically significant relationship between boss area versus disc area (Boss Area = 26.2 + 0.041 Disc Area), that accounts for 19% of the observed variability. Although there is significant scatter within the dataset the relationship is greater than would be expected by chance alone (Fig 4). Linear regressions when separated on geographic basis generally result in better linear regression fits. The Hellefjord disc and boss dimensions closely match the relationships seen in discoidal fossils at Ferryland, within the Ediacaran Fermeuse Formation, consistent with a similar positive hyporelief preservation style. These Fermeuse Formation fossils are dominantly smaller than those from other fossil bearing surfaces from the Ediacaran of Newfoundland (Fig 5). Hence, the morphology of the Hellefjord discoidal fossils, size, distribution and relationship to stem like features, are similar to descriptions of holdfasts from the Ediacaran System (Fig 6). However, we note that successful bodyplans, able to remain structurally stable, can only have a limited range of stem to holdfast dimensions dependant on an array of factors, including but not limited to substrate stability, current velocity, stem length, and size of frondose element. Based on the disc and boss dimensions, we suggest that a bodyplan with general similarity to forms of Ediacaran age developed in the Hellefjord Schist under comparable environmental conditions, given the analogous depositional setting.

It is important to note that this interpretation should not be taken as evidence of any direct or meaningful close biological relationship between Silurian and Neoproterozoic forms. Frondose morphology independently evolved at least three times in the Ediacaran, in the rangeomorphs, arboreomorphs, and erniettomorphs, and perhaps more often if other attached epibenthic taxa such as *Thectardis* are considered [109,110]. In the Phanerozoic, several additional groups independently evolved a similar morphology, most notably the octocorallid cnidarian ‘sea pens’ (previously suggested as an affinity for some fronds of Ediacaran age, though subsequently ruled out; see [111], and also graptoloids, pelmatozoan echinoderms (blastoids and crinoids), poriferans, actinians, algae, and others. Whilst not all of these organisms are unmineralized, and whilst some are adapted for hard rather than soft substrates, this still strongly indicates that frondose morphology is relatively easy to attain through convergent adaption to similar environments.

The question of the biological affinities of these fossils is impossible to answer with the material presented herein. Without a well-preserved upper part that can be definitively linked to a specific group, it is not possible to assign these fossils with any degree of confidence. Additionally, given the significant age difference of these fossils to other comparable forms, it is entirely feasible that they represent a different, previously unknown group, which evolved independently to attain a similar form due to evolutionary convergence. Hence, no primarily biological conclusions should be drawn.

Instead, we contend that these fossils are primarily indicative of environmental and ecological conditions. The frond-holdfast nature of these Silurian fossils is not of particular significance in isolation, given that numerous groups have independently evolved such a bodyplan. Rather, the significance of the Hellefjord fossils, and the justification for comparison to frondose specimens of Ediacaran age, lies in the combination of the unmineralized nature, the frond-holdfast morphology, and perhaps most importantly, in the nature of attachment of these unmineralized frond-holdfast organisms to the substrate. Phanerozoic frondose organisms are generally attached to hard substrates by means of root-like structures, or anchored in soft substrates by means of a deep, bulbous peduncle. Frondose forms of Ediacaran age, by contrast, were anchored on soft firmground substrates by means of a discoidal holdfast, a feature that has not previously been described in any subsequent frondose organism. The unmineralized Hellefjord frond-holdfast fossils similarly appear to have anchored by means of a discoidal holdfast; by far the youngest example of such a bodyplan.

Many factors have been proposed to control the Ediacaran-Cambrian diversification of animals, along with the origin of biomineralisation and the substrate changes in the early Cambrian, referred to as the Cambrian Substrate Revolution [112] or the Agronomic Revolution [100]. Some factors link to the importance of environmental and preservational change, others support animal developmental innovations, while another suite of explanations focuses on the growth of new ecological relationships [113]. It is likely that the events of the Ediacaran and Cambrian involved all of these factors [114]. A particular concern with regard to discoidal fossils has been to find a satisfactory explanation for their apparent restriction to the late Neoproterozoic. Proposals to address the apparent stratigraphic restriction included suggestions that some organisms during the Ediacaran were constructed from unusually tough biological materials to account for their preservation [115]. Specifically, such robust construction was seen as a means for the preservation of forms like *Dickinsonia* recorded as positive epirelief moulds of negative hyporelief casts. More recently, burrowing was proposed to have expunged the microbial mats necessary for the preservation of soft bodies in marine environments [103,116]. Specifically, vertical burrowing, which may have evolved as a defence against predation, has been widely proposed to have opened up new ecological niches beneath the sea floor as water and oxygen could now get into deeper sediment layers. At the same time, and consequentially, microbial mats were progressively destroyed and forced into more restricted habitats, in environments unfavourable for animals. This change in substrate is thought to be partly responsible for the demise of the ecological niches that the frond-holdfasts organisms (and, others) of the Ediacaran occupied [112, 117].

Importantly, the observations in this work indicate that discoidal impressions with forms ostensibly identical to some biological structures of Ediacaran age occur in Llandovery sediments, rendering the stratigraphic requirement for such explanations moot, while supporting the nature of substrates as a primary environmental and ecological control on the distribution of organisms with particular morphologies. We consider the most likely explanation for the similarity of the Hellefjord discs in bodyplan to organisms of Ediacaran age is convergent adaptation of both the overall unmineralized frond-holdfast bodyplan, and the attachment to the substrate by means of a discoidal holdfast, to similar environmental and ecological (including substrate) conditions.

## Conclusions

Discoidal features preserved within the mid-Llandovery (Lower Silurian) Hellefjord Schist are interpreted as fossils. These fossils have morphological and taphonomic similarity to Neoproterozoic forms elsewhere, including a similar relationship between central boss (stem attachment) and disc area. The Hellefjord Schist is intruded by 438 ± 2 Ma granites and contains a young volcaniclastic zircon population that constrains deposition of the unit and its fossil assemblage to between 437 ± 2 and 439 ± 3 Ma. This geochronology allows us to demonstrate that the Hellefjord fossils represent an early Silurian organism, similar in general morphology to Neoproterozoic frond-holdfast organisms such as the arboreomorphs. However, rather than suggesting any close biological relationship, we contend that the occurrence of these fossils indicates that their habitat was similar enough to Ediacaran environments that a frond-holdfast bodyplan was a viable strategy, leading to a similar morphology developing through convergent adaptation to both the overall environment and the nature of the substrate. Previously, it has been argued that the Cambrian Substrate Revolution removed the ecological niches that the frond-holdfasts organisms (and, others) of Ediacaran age occupied. The observations from the Hellefjord Schist show that this kind of niche environment still existed a hundred million years later. The occurrence of fossils attributable to *Nimbia* and *Tirasiana* in post-Ediacaran rocks confirms that the presence of apparently characteristic depauperate Ediacaran-like fossils cannot be used unambiguously as evidence of Neoproterozoic age [48].

Finally, it is important to note that these discoidal fossils and apparent stems, although imperfectly preserved, have nonetheless been retained within rocks metamorphosed to amphibolite facies. This demonstrates that even high-grade metamorphic recrystallization will not always remove all evidence of unmineralized organisms. Strata which have not previously been systematically explored for such unmineralized fossils due simply to their metamorphic grade may therefore represent an important untapped source of information about the ancient biosphere.

## Supporting Information

S1 Table Zircon U-Th-Pb data table. U-Th-Pb SIMS results from zircon grains.
(XLS)

S1 Text U-Th-Pb method. Supplimentary U-Pb geochronology method information.
(DOCX)

S1 Video Photogrammetric reconstruction of fossils. Movie file of a 3D photogrammetric reconstruction of discoidal fossils in the Hellefjord Schist, Northern Norway.
(ZIP)
