# Supplementary material for: Cryptic Disc Structures Resembling Ediacaran Discoidal Fossils from the Lower Silurian Hellefjord Schist, Arctic Norway
Source: PLoS One. 2016 Oct 26;11(10):e0164071. doi: 10.1371/journal.pone.0164071 (PMC5082646; doi:10.1371/journal.pone.0164071)
Supplement: S1 Text — Supplimentary U-Pb geochronology method information. (DOCX) [file pone.0164071.s002.docx]

S1 Text; **U-Th-Pb method**

Zircon separation was performed by gold panning sieved and crushed samples (grain size <500 µm). Zircon crystals were hand picked from the panned heavy concentrate using a binocular microscope and mounted in an epoxy resin disc along with the zircon standard 91500. This epoxy mount was polished to half grain thickness to reveal the crystal interiors, gold-coated and imaged with an SEM, using a cathodoluminescence (CL) detector to reveal internal structures. U–Th–Pb zircon analyses were performed using a Cameca IMS 1270 ion microprobe, following methods described by Whitehouse et al. (1999). U/Pb and concentration calibration was based on analyses of the Geostandards zircon 91500 (Wiedenbeck et al. 1995). Analytical data are presented in DR3 with analytical errors shown at the 1σ level. Age calculations were made using Isoplot version 3.02 (Ludwig 2003). Where common lead correction has been applied (DR3) a modern-day average terrestrial common Pb composition is assumed (Stacey & Kramers 1975).

Ludwig, K.R. 2003. User’s Manual for Isoplot 3.00, a Geochronological Toolkit for Microsoft Excel. Berkeley Geochronology Centre Special Publications, 4.

Stacey, J.S. & Kramers, J.D. 1975. Approximation of terrestrial lead isotope evolution by a two-stage model. Earth and Planetary Science Letters, 26, 207–221.

Whitehouse, M.J., Kamber, B.S. & Moorbath, S. 1999. Age significance of U–Th–Pb zircon data from Early Archaean rocks of west Greenland: a reassessment based on combined ion microprobe and imaging studies. Chemical Geology (Isotope Geoscience Section), 160, 201–224.
